# Malignancy rate of 10,731 uteri morcellated during laparoscopic supracervical hysterectomy (LASH)

**DOI:** 10.1007/s00404-015-3696-z

**Published:** 2015-03-28

**Authors:** Bernd Bojahr, Rudy Leon De Wilde, Garri Tchartchian

**Affiliations:** 1 Klinik für Minimal Invasive Chirurgie, Kurstraße 11, 14129 Berlin-Zehlendorf, Germany; 2 Clinic of Gynecology, Obstetrics and Gynecological Oncology, University Hospital for Gynecology, Pius-Hospital Oldenburg, School of Medicine and Health Sciences, Carl von Ossietzky University, Oldenburg, Germany

**Keywords:** Laparoscopic supracervical hysterectomy, Morcellation, Uterine sarcoma, Endometrial cancer

## Abstract

**Purpose:**

This study aims to evaluate the number of cases of occult uterine malignancies in all LASH surgeries at the MIC clinic (Berlin) and to verify how the operative technique affects the prognosis of the disease.

**Methods:**

Data of 10,731 patients who underwent a standardized LASH surgery with electric power morcellation between 1998 and April 30, 2014 were retrospectively analyzed. Main indication for LASH was symptomatic uterine myomas (81.3 %).

**Results:**

No intra-operative complication was caused by use of a morcellator. In total, six sarcomas (0.06 %), including four endometrial stromal sarcomas (0.04 %) two leiomyosarcomas (0.02 %), and eight endometrial cancers (0.07 %) were documented. This amounts to a very low uterine malignancy rate of 0.13 %. Median follow-up period for all six patients with sarcoma and seven patients with endometrial cancer was 65.58 months (13–169). No recurrence was reported for the patients with endometrial cancer and five sarcoma patients in the comprehensible follow-up period. One patient died 13 months after LASH surgery due to the diagnosed leiomyosarcoma with peritoneal carcinomatosis and bone metastases.

**Conclusion:**

In 10,731 morcellated uteri during LASH only 0.06 % sarcoma and 0.07 % endometrial carcinoma were detected. All patients should be informed about the rare possibility of a malignant disease during pre-operative counseling. With a timely follow-up surgery according to the oncologic guidelines, our data suggest a very good prognosis in terms of survival after LASH with morcellation of malignant tumors in the uterus.

## Introduction

In present-day gynecology, hysterectomy belongs to the standard procedures and is one of the most commonly performed surgeries. The first laparoscopic hysterectomy was performed by Harry Reich [1] 25 years ago in 1989. Since 1990 the laparoscopic supracervical approach to hysterectomy has evolved both in Europe and the US [2–5].

Advancements of endoscopic instruments and of operational techniques have meanwhile led to a significant reduction in the number of abdominal hysterectomies. This is because more vaginal hysterectomies in combination with laparoscopy, showing a lower rate of complications, are performed [6, 7].

During laparoscopic assisted vaginal hysterectomy (LAVH) the uterus can be morcellated and removed vaginally after respective laparoscopic preparation. Without indications for removal of the cervix, the cervical stump can be retained and the corpus uteri is removed via laparoscopic morcellation using an electronic morcellator during laparoscopic supracervical hysterectomy (LASH). Because of this procedure, the necessity for abdominal hysterectomy very rarely arises at the MIC clinic Berlin, where even large uteri can be removed using minimally invasive techniques.

However, the American Association of Gynecologic Laparoscopists (AAGL) stated that, in case of known or suspected uterine malignancy, morcellation is contraindicated [8]. Due to safety concerns—more specifically, the potential for dissemination of occult uterine cancer that may occur via morcellation during hysterectomy and myomectomy leading to a poorer prognosis—the Food and Drug Administration (FDA) recently issued a statement discouraging the use of electromechanical morcellation for hysterectomy and myomectomy in most women with uterine myoma [9].

As patient safety and patient satisfaction are the main goals of all new developments in endoscopic surgery, the AAGL convened a tissue extraction task force to examine the propriety of this ban. Based on existing literature, the data concerning the frequency of malignancies during morcellation needs to be updated in order to confirm that the use of morcellation during hysterectomy and myomectomy does not offer a significant patient disadvantage versus open surgery in the rare occurrence of malignancy being discovered post-operatively or for the development of new concepts to improve patient safety [10].

Endometrial cancer is the most common gynecologic malignancy in the US, with over 50,000 new cases and almost 8600 related deaths each year [11]. The median age of diagnosis is 66 and very often abnormal bleeding is a first sign of the disease [12]. Leiomyosarcomas and other uterine sarcomas are very rare, accounting for up to 7–8 % of all uterine cancers [13]. The median age at which uterine leiomyosarcoma is diagnosed is usually between 47 and 56 years [14].

The aim of this study was to evaluate the number of cases of occult uterine malignancies in all LASH operations at the MIC clinic and to verify how the operational technique affects the prognosis of the disease by tracking all patient files.

## Materials and methods

### Patients and data collection

In a retrospective study, the number of cases of occult uterine malignancies in LASH operations was evaluated for the period from 1998 to April 30, 2014. During this time, a total of 10,731 LASH surgeries were performed at the MIC clinic. The exact figure of malignant uterine findings during LASH surgery was available due to an inaugural dissertation from 2005 which recorded all histological findings of 1706 LASH surgeries performed between 1998 and October 2003 [15]. Therefore, patient data for all patients with malignancy till October 2003 could be collected and analyzed as currently valid. From November to December 2003 no malignant findings were recorded in any LASH surgery. In 2004 a questionnaire for a mutual cancer registry was introduced, which obliges every clinic in 6 German federal states to report each malignant finding. Therefore, all cases of endometrial carcinoma, uterine sarcoma, including leiomyosarcoma, were available for the entire period of this study. All patient files were collected. Via telephone the treating gynecologist was asked about the time of the last examination, possible recurrences and therapies of the patient.

Our data relate to endometrial stromal sarcoma, endometrial cancer, leiomyosarcoma and carcinosarcoma. Smooth muscle tumors with uncertain malignant potential [atypical leiomyomas, smooth muscle tumors of undetermined malignant potential (STUMP), proliferative leiomyomas and high-grade proliferative leiomyomas] are not recorded in the cancer registry and were not evaluated before 2004 [15]. Therefore, these tumors could not be evaluated in this retrospective study. Findings concerning atypical myomas and tumors of undetermined malignant potential were of incidental nature. Three cases, reported in this study, concerning atypical myomas and tumors of undetermined malignant potential are only identified due to further documented follow-up surgery as part of different surveys or later documented malignancy. They are also included in the discussion. No Institutional Review Board (IRB) approval was obtained.

Main indication for LASH were the symptomatic uterine myomas (81.3 %) [7, 16].

Premalignant or malignant findings in the area of the cervix and the corpus uteri were considered as contraindications for laparoscopic supracervical hysterectomy.

All patients were pre-operatively vaginally examined by the surgeon including a speculum of the cervix and a vaginal sonography was also conducted. In case of sonographic endometrial abnormalities, a diagnostic hysteroscopy and endometrial curettage was performed before LASH. The endometrial thickness was not obligatory documented. A cervical PAP test, not older than 1 year, was available for every patient.

### Surgical procedure

The previously published LASH operation technique with electric power morcellation was standardized and remained consistent throughout the observation period [7, 16, 17]. Surgery was performed by 4 surgeons. High patient satisfaction [7–9], low complication rates [7, 16] and very low conversion rates to abdominal hysterectomies have already been reported [7, 16, 17].

### Pathology and follow-up

The material was immediately fixed in 7.5 % buffered formaldehyde. After fixation for minimally 24 h all cores were macroscopically examined by two experienced gynecological pathologists according to color, consistence, necrosis and margins. All suspicious cores were separated. Photo documentation was done.

Cut sections (4–5 μm) were stained in hematoxylin–eosin in a fully automatic stainer (Symphonie Fa Roche). Microscopy was done using an Olympic BX51.

Immunohistochemistry was obtained by using the Bench Mark XT (Fa Roche/Ventana). All sarcoma and diagnoses were re-examined and confirmed in a German pathologic reference-center.

The key diagnostic microscopical criteria for leiomyosarcomas were proliferative or mitotic activity, nuclear and cytological atypias and findings of geographic necroses. In case of endometrial stromal sarcoma, enrichment of small uniform cells was detected microscopically. Endometrial stromal morphology was still discernible and resembles proliferation phase stroma. Significant atypias or conspicuous pleomorphism was not detectable. In case of endometrial stromal sarcoma CD 10 was found positive during immunohistochemistry.

Potentially malignant myomas were defined as epithelial leiomyomas larger than 6 cm. Less than 4 mitoses/10 HPF (high power fields) and mild atypias may happen.

### Statistics

For the parameters age and uterus weight the mean was calculated. Data were analyzed using Windows-Excel (Microsoft 2010). Mean values were calculated and shown with standard deviations and 95 % CI.

## Results

### Malignancies at time of surgery

Patient data from 10,731 LASH operations with electric power morcellation were evaluated. The indication for surgery in 8720 patients (81.3 %) was uterine myomas, in 1015 patients (9.4 %) bleeding disorder, in 361 patients (3.4 %) suspected uterine adenomyosis and in 635 patients (5.9 %) uterine prolapse. In case of uterine prolapse LASH was combined with a sacropexy of the cervical stump.

No intra-operative complication was caused by the use of the morcellator. In total, six sarcomas (0.06 %), including two leiomyosarcomas (0.02 %) and eight endometrial cancers (0.07 %) were documented. For detailed data see Table 1.

**Table 1:** Malignant histological uterine findings after LASH

	*n*	%
Uterine malignancies	13	0.13
Sarcoma	6	0.06
Low-grade endometrial stromal sarcoma	4	0.04
Leiomyosarcoma	2	0.02
Endometrial cancer	8	0.07
Endometrial cancer in situ	1	0.01
Adenocarcinoma located in a polyp	1	0.01
Undifferentiated endometrial carcinoma	1	0.01
Endometrioid adenocarcinoma	5	0.05

Out of the six sarcomas, four patients exhibited a low-grade endometrial stromal sarcoma.

In two patients a leiomyosarcoma was detected. Both patients with leiomyosarcoma were 49 years old and presented significantly enlarged uteri, weighting 1.000 and 567 g, respectively.

Median age, weight and height of all patients with uterine malignomas including endometrial cancer and sarcomas can be found in Table 2.

**Table 2:** Demographic data of patients with endometrial cancer and sarcomas

	Endometrial cancer	Endometrial stromal sarcoma	All uterine malignomas
Patients age			
Mean years ± SD	51.4 ± 6.1	48.7 ± 5.7	50.2 ± 5.8
Range	51.6–53.2	48.5–49.9	50.1–51.1
Patients height			
Mean cm ± SD	171.4 ± 7.0	168.8 ± 3.5	170.4 ± 5.7
Range	171.3–175.3	168.7–173.1	170.3–173.2
Patients weight			
Mean g ± SD	88.9 ± 31.6	75.2 ± 12.9	83.0 ± 25.6
Range	88.2–90.8	74.8–77.1	82.6–84.4

In 8720 cases of diagnosed uterine myomas serving as indication for LASH surgery only 4 patients were ultimately found to have a histological proven sarcoma: this amounts to a sarcoma rate of 0.05 %. In two patients bleeding disorders were the indication for LASH. One month before LASH one of these two patients underwent hysteroscopy and endometrial curettage without any sign of malignancy. The second patient did not undergo a hysteroscopy and endometrial curettage due to unsuspicious findings during pre-operative endometrial sonography.

Eight endometrial cancers were found, one endometrial cancer in situ (a noninvasive endometrial lesion with serous differentiation), one adenocarcinoma, located in an endometrial polyp, one undifferentiated endometrial carcinoma and five endometrioid adenocarcinomas. However, except for the undifferentiated endometrial carcinoma, the tumor was in an early stage (stage I—FIGO—UICC).

### Recurrences and follow-up surgery

Follow-up data for all six patients with sarcoma and seven out of eight patients with endometrial cancer are available. Median follow-up period was 65.58 months (13–169). All patients with sarcoma and six patients with endometrial cancer underwent re-exploration.

There is no data available for the patient with endometrial cancer in situ with regard to recurrences, follow-up surgery or death. No recurrence was reported for the remaining seven patients with endometrial cancer in a median follow-up period of 74 months (16–169 months).

For seven patients with endometrial cancer, as documented above, they underwent follow-up surgery 32 days (11–56 days) after LASH. In all seven patients a cervical extirpation with bilateral salpingo-ovarectomy and a “pelvic wash” was performed: no residual tumor or tumor cells were found (see Table 3).

**Table 3:** LASH and endometrial carcinoma

Patient	Patients age (years)	Body mass index	Date of operation	Uterus weight (g)	Histology	Last gyn. examination	Follow-up (months)	Recurrence
1	57	43.6	16.03.2000	300	Adenocarcinoma in polyp, pT 1a	04/2014	169	No
			27.03.2000		No tumor in cervix, adnexa, lavage			
2	60	29.7	25.01.2001	84	Endometrioid adenocarcinoma G1	07/2013	151	No
			12.02.2001		No tumor in cervix, adnexa, lavage, pT 1b			
3	50	21.6	26.09.2000	150	Endometrial cancer in situ, pT 1a	No information available		
4	45	19.7	13.05.2004	400	Undifferentiated endometrial cancer, pT 1a/b, N1 (5/17), MX, V0, L0; FIGO III C	06/2009	61	No
			03.06.2004		No tumor in cervix, adnexa, 5 positive lymph nodes			
5	46	27.9	29.07.2008	157	Endometrioid adenocarcinoma pT1a G2	11/2009	16	No
			15.08.2008		No tumor in cervix and 20 lymph nodes	Moved to another city, lost to follow-up		
6	46	50.0	03.12.2008	230	Endometrioid adenocarcinoma, pT1a G1–2	05/20013	53	No
			02.01.2009		No tumor in cervix, adnexa, lavage and 28 lymph nodes			
7	58	29.6	01.03.2001	50	Endometrioid adenocarcinoma, pT1b G1	05/2014	38	No
			12.04.2011		No tumor in cervix, adnexa, lavage			
8	49	20.7	06.12.2011	26	Endometrioid adenocarcinoma, pT1a G1	05/2014	30	No
			31.01.2012		No tumor in cervix, adnexa, lavage			

Two patients underwent an additional pelvic lymphadenectomy (see Table 3). In one patient 20 nontumorous lymph nodes were removed. In the patient with undifferentiated endometrial carcinoma five lymph node metastases were found. This patient received further treatment in an oncological center. The latest medical information about this patient is at 61 months post-LASH: at this time the patient was free from recurrence. The latest medical information about the patient with the 20 nontumorous lymph nodes is at 16 months post-LASH: no signs of recurrence were present at that time (see Table 3).

Follow-up surgery in case of sarcoma was done 51 days (2–91) after LASH: no residual tumor or tumor cells were found. In one obese patient with leiomyosarcoma a peritoneal carcinomatosis was detected during laparotomy 10 months post-LASH. This patient died 13 months after LASH surgery due to the diagnosed leiomyosarcoma with peritoneal carcinomatosis and bone metastases. Two months after LASH this patient underwent a laparoscopic cervical stump extirpation without intra-operative signs of metastasis. Eleven months after primary surgery peritoneal metastasis and bone metastases were detected radiologically.

One patient with leiomyosarcoma (follow-up of 137 months) and four patients with endometrial stromal sarcoma were still alive when last information was available with a follow-up period of 22, 57, 36 and 36 months, respectively. All patients underwent follow-up surgery: no histological abnormalities were detected in the extirpated cervix and adnexa. The patient with endometrial cancer and additional stromal sarcoma also underwent cervical and adnexal extirpation: no histological abnormalities were detected, and 3 years after operation no recurrences were present. For the past 3 years there is no further information available concerning this patient. In one patient with endometrial stromal sarcoma no signs of recurrence were detected 26 months post-surgery. From May 2010 onwards there is no further information available with regard to this patient. See also Table 4.

**Table 4:** LASH and uterine sarcoma

Patient	Patients age (years)	Body mass index	Date of operation	Uterus weight (g)	Histology	Last gyn. examination	Follow-up (months)	Recurrence
1	49	27.4	04.12.2002	567	Leiomyosarcoma	05/2014	137	No
			06.12.2002		No tumor in cervix, adnexa, lavage			
2	44	22.1	25.03.2008	106	Endometrial stromal sarcoma (low grade)	05/2010	26	No
			14.07.2008		No tumor in cervix, adnexa, lavage			
3	43	29.0	17.06.2008	590	Endometrial stromal sarcoma (low grade)	03/2014	57	No
			02.10.2008		No tumor in cervix, adnexa lavage			
4	48	21.2	13.05.2008	90	Endometrial stromal sarcoma (low grade)	05/2011	36	No
			12.08.2008		No tumor in cervix, adnexa, lavage			
5	49	33.7	04.11.2008	1000	Leiomyosarcoma		13	Yes
			07.10.2009		Peritoneal carcinomatosis and bone metastases			Death 12/2009
6	59	24.8	15.01.2009	440	Endometrial stromal sarcoma	12/2012	36	No
			26.02.2009		No tumor in cervix, adnexa, lavage			

Besides above malignant findings, histological abnormalities were detected in three other patients. In 2001 a uterus weighting 180 g was removed in a 51-year-old patient: histological examination revealed a mesenchymal neoplasia, a UTROSCT-tumor (uterine tumor resembling ovarian sex cord tumor). During removal of the cervix 6 months later no remnants of the tumor were detected. At the time of follow-up examination in January 2013 the patient was free of recurrence.

No further data are available for one patient with a 450 g uterus with potential malignant myoma after February 2002: 17 months post-surgery this patient was free from complaints.

In 2001 one 55-year-old patient with a 230 g uterus was diagnosed with high-grade proliferative myoma, a benign, cellular leiomyoma without necrosis or mitotic activity <10 per 10 HPF. Due to abdominal problems she underwent a laparoscopy in 2012 with tumor extirpation at the anterior abdominal wall. Histology revealed a benign endometrial stromal tumor based on the type of tumor margin. Five months later in March 2013 (12 years post primary LASH), a recurrent tumor was removed and a low-grade endometrioid stromal sarcoma was detected histologically. This tumor represents a completely different entity than the one detected in 2001 and it remains questionable if both are related. In May 2014 (14 months after diagnosis) and after a letrozol therapy this patient was free of recurrence and complaints.

## Discussion

The presented data show a very low uterine malignancy rate in patients undergoing LASH, with the most aggressive tumors with a bad prognosis occurring even less commonly. Leiomyosarcoma is a very aggressive and rare cancer and only has a small part in the group of malignancies in LASH. This and other uterine sarcomas account for up to 7–8 % of all uterine cancers [13]. The FDA estimates that 0.3 % of all women undergoing hysterectomy or myomectomy for the treatment of fibroids are found to have an unsuspected uterine sarcoma [9]. In our study only six sarcomas [0.06 % ± 2.4 (0.054–0.057)], including two leiomyosarcomas [0.02 % ± 1.4 (0.018–0.019)], were found. Correspondingly the probability of accidently morcellating a sarcoma is very low. Theben et al. found an unexpected malignancy rate of 0.25 % (*n* = 4 out of 1584 patients) after LASH. Two patients were diagnosed with endometrial cancer and leiomyosarcoma. Yet in the short period of the study (28–52 months) these patients remained free of recurrence after treatment [18].

Five out of six patients with sarcomas are still alive with a follow-up period of 58.4 months. To our knowledge, only one patient with a leiomyosarcoma died 13 months after surgery. The progression-free survival in leiomyosarcoma is normally 1.1 years, median survival is 3.6 years and 5-year survival rates for stage I tumors is 57 % for leiomyosarcoma [19]. Thus the death of one of our patients with leiomyosarcoma confirms the bad prognosis associated with this tumor. However, it is questionable which effect morcellation has on the progress of the disease or which progress would have been observed after abdominal hysterectomy or no operation in case of an enlarged uterus weighting over 1000 g.

In case of endometrial cancer, no related death or recurrence after LASH with morcellation of the uterus and a timely follow-up surgery was found. Only the post-surgical data of one patient with endometrial cancer, in fact the one patient with the best prognosis of all patients, is missing. In summary our data suggest a very good prognosis in terms of survival after LASH with morcellation of malignant tumors in the uterus, if a timely follow-up surgery according to the oncologic guidelines is performed.

The four patients with stromal sarcoma had a very good prognosis. Although there are only few cases in summary our results show better prognosis in case of endometrial stromal sarcoma compared to leiomyosarcoma.

Despite preliminary examination, including vaginal sonography, eight endometrial carcinomas were only detected after the histopathological examination of the LASH specimen. In case of suspicious sonography during pre-operative diagnostic investigation diagnostic hysteroscopy and endometrial curettage was conducted before LASH.

Four patients with endometrial carcinomas underwent an endometrial curettage and diagnostic hysteroscopy before LASH and showed no signs of malignancy. This demonstrates that due to known error rates even for curettage and sonography there will always be individual cases with unexpected malignancies [20–23].

In this retrospective study, all morcellated uterine myomas occurring in the study period were recorded. As smooth muscle tumors with uncertain malignant potential (atypical leiomyomas, smooth muscle tumors of undetermined malignant potential (STUMP), proliferative leiomyomas and high-grade proliferative leiomyomas) were not included in this study, patients with undetected pre-malignancies are probably left out unconsidered. These patients might expect recurrences.

No data are available concerning the effect of electric morcellation on recurrence and probable formation of sarcomas in case of these tumors. As indicated by this study and further literature these cases should be systematically evaluated and re-examined. If possible the progress should be recorded in specific registries in the same manner as malignant diagnoses are already documented.

STUMP-tumors have a low recurrence rate, ranging from 7.2 % [24] to 27 % [25]. However, there is the possibility that despite their uncertain nature, STUMPs can exhibit metastatic activity, recur as STUMPs or undergo malignant transformation to leiomyosarcomas [24, 26]. Therefore, in all cases of histological proven atypical myomas or STUMP-tumors a diagnostic laparoscopy either in case of clinical signs of recurrence or at the latest 1 year post-surgery, is recommended at the MIC clinic. In the few cases 1 year post-surgery a diagnostic laparoscopy was performed to exclude tumor recurrence. However, in our study no recurrence was reported after LASH with preceding atypical myomas or STUMP-tumors. In most cases these tumors are benign. At present day there are no factors known to predict the risk of recurrence in those entities.

All patients need to be informed about the rare possibility of a malignant disease and alternative treatment methods during pre-operative counseling. Recurrence and long distant metastases are significantly rarer. However, they develop much later as in case of leiomyosarcomas, which occur soon after surgery. Recently a retrospective, multi-institution study of patients with stage I–II uterine leiomyosarcoma whose uteri were removed intact, found recurrence in the first 2.5 years after diagnosis in 71.8 % of all patients [27]. Even 20 years post-surgery recurrent disease is possible [28]. In case of post-operative malignancy finding, a timely follow-up surgery according to the oncologic guidelines is necessary. Essentially in case of such rare incidences, the prognosis is difficult to predict.

Only after applying an abundant lavage at the end of laparoscopy after removal of all abdominal remnants of myomas and tissues and careful control of the area of operation as well as the middle and upper abdomen the surgery is finished. This way the risk of complications and bleedings and remaining tissue in the abdomen are tried to be excluded.

Due to the lower malignancy rate shown in our study as compared to the data presented by the FDA, we conclude that the abandonment of morcellation is not necessary. The need for morcellation of every uterus in retrieval pouches still has to be proven in further studies.

The FDA discouraged the use of laparoscopic morcellation for hysterectomy and myomectomy [9] based on very few mostly single-institution and retrospective studies (8 publications) with an inadequately low number of patients (more than half contain fewer than 1000 patients). In our view along with the task force’s analysis [10] the malignancy rate, to be actually expected, during morcellation of uteri or myomas, derived from these studies, is inadequately proven.

It is of positive value that the FDA has put this problem on the agenda. However, the consequence of this recommendation for all patients with myomas disregards many of the scientific proven advantages of minimal invasive surgery.

The use of specimen retrieval pouches represents an option; it is however unclear to decide which cases are intra-operatively doubtful, or this should be a general measurement. Also further research on the use of specimen retrieval pouches during morcellation is necessary to examine the possibly increasing complication rate concerning injuries to adjacent organs. The Task force states: “The use of morcellation within specimen retrieval pouches for containment of benign or malignant uterine tissue requires significant skill and experience, and the use of specimen retrieval pouches should be investigated further for safety and outcomes in a controlled setting” [10].

All patients need to be informed about the rare possibility of a malignant disease despite accurate pre-operative evaluation without signs of malignancy or pre-malignancy, during pre-operative counseling. Additionally the necessity of follow-up surgery, depending on the findings, has to be pointed out. In case of a timely second operation, the prognosis is shown to be very good. Only for a few patients, in case of leiomyosarcomas, prognosis is very bad; however, this prognosis is generally very bad, even with abdominal hysterectomy and no morcellation. Tumor stage is the most important prognostic factor for all histological types, with 5-year overall survival being 50–55 % for stage I and 8–12 % for stage II–IV disease [29, 30].

Unfortunately in these cases no pre-operative diagnostics are known to be predictive of the subsequent histological findings.

Although our results confirm the rare occurrence of uterine sarcomas, improved diagnostic methods are necessary to distinguish between malignant and benign myomas, as recommended by the AAGL. Furthermore, criteria for the identification of high-risk groups must be developed. This is only possible through data evaluation from national and international cancer and sarcoma registries and prospective and retrospective multicenter studies to examine the prevalence and progression after different treatment regimens, allowing a central evaluation of the treatment outcomes in uterine sarcomas.

In the 195th statement the German Association for Gynecology and Obstetrics (DGGG) quote an incidence rate of uterus sarcomas of 1.32 in 100,000 women in Germany in 2010 and of 1.3 in 100,000 woman in Bavaria between 2002 and 2011 [31] for gynecologic morcellators.

Furthermore the DGGG emphasizes in this statement, that all patients should be sufficiently informed about the potential risk, advantages, disadvantages and alternatives (non-operative, operative) during pre-operative counseling [31].

The German Society for Gynecological Endoscopy (AGE) underlines that in many cases electric morcellation is important for the treatment of symptomatic myomas. This way the favorable treatment via minimally invasive laparoscopic hysterectomy or organ-conserving myomectomy instead of abdominal hysterectomy is possible in many cases. Banishment of electric morcellation would lead to a higher morbidity and mortality. However, all patients have to be informed about the low probability of malignant disease [32].

## Conclusion

A very small incidence of sarcoma (0.06 %) and endometrial carcinoma (0.07 %) were detected in 10,731 uteri morcellated after LASH. All patients should be informed about the rare possibility of a malignant disease during pre-operative counseling.

With a timely follow-up surgery according to the oncologic guidelines our data suggest a very good prognosis in terms of survival after LASH with morcellation of malignant tumors in the uterus. Surgeons should not miss the advantages of morcellation during laparoscopic surgery given that there are no pre-malignancies or malignancies detected pre-operatively.
